# A Dataset of Benchmark Boolean Models for Gene Regulatory Networks

**DOI:** 10.1038/s41597-026-07585-6

**Published:** 2026-06-06

**Authors:** Caya L. O. Hotstegs, Jose P. Llano, Hans A. Kestler

**Affiliations:** 1 Institute of Medical Systems Biology, Ulm University, Albert-Einstein-Allee 11, 89081 Ulm, Germany; 2 Department of Mathematics, University of Castilla-La Mancha, 02071 Albacete, Spain; 3 Leibniz Institute on Aging - Fritz Lipmann Institute, Beutenbergstraße 11, 07745 Jena, Germany

## Abstract

Gene regulatory networks (GRNs) capture the processes involved in gene regulation. Boolean network (BN) modeling provides a simple but effective framework for understanding the dynamical behavior of GRNs. Although BNs have been widely studied and applied, algorithms and theoretical analyses are usually tested on ad hoc selected or artificially constructed models, which may introduce bias and fail to capture the essential structural and dynamical properties of real GRNs for which they are ultimately intended. Benchmarking offers standardized models for validation and comparison of computational methods and analyses. We construct benchmark BN models for GRNs of four major biological kingdoms: animals, bacteria, fungi, and plants. All models are built from empirically observed recurrent properties and motifs in GRNs. The proposed benchmark BNs provide a systematical and unbiased basis for evaluating algorithms and theoretical analyses.

## Background & Summary

Modern systems biology requires us to move beyond the study of isolated gene interactions. Gene regulatory networks (GRNs) capture the complex mechanisms through which gene regulation occurs and can provide essential knowledge of system-level properties. Their proper study is facilitated by the use of mathematical network models.

Boolean networks (BNs) are qualitative mathematical models that are used to represent GRNs. Originally introduced by Kauffman^1^, they have since become one of the most popular and powerful tools for analyzing GRNs^2–8^, and biological systems in general^9–11^. In a BN, each node in the network graph represents a biological entity, and the directed edges between them account for their influence, either activating (represented by a positive signed edge) or inhibiting (represented by a negative signed edge). Each node has a binary state, either on (1) or off (0) at a given time. A local Boolean function updates the state of each node based on the states of its neighbor nodes. The iterative application of all local functions defines the dynamics of the system whose analysis reveals properties of its long-term behavior. Specifically, the attractors of the system correspond to its stable configurations, which are often associated with phenotypes.

The analysis of the dynamics of BNs has grown into a research field of its own, with countless works focusing on the study of their theoretical^12–16^ and computational properties^5,17–20^. A proper evaluation of many theoretical and computational studies requires testing on real GRNs. However, testing most often relies on arbitrarily chosen (or even ad hoc) BN models of GRNs. This can introduce bias, since such models may miss common properties and motifs appearing in real-world BN models of GRNs. Moreover, the large number of available BN models of GRNs makes it challenging to select a representative subset that covers the diverse structural properties without over- or under-representation.

Benchmark models are constructed from common properties and provide a standard framework for algorithmic testing and theoretical analyses, ensuring reproducibility and enabling practical comparison of results across studies. Specifically, research has revealed properties and motifs common in real-world BN models of GRNs. These include the scale-freeness for out-degree distribution^21,22^, the appearance of feed-forward loops (FFLs) and feedback loops (FBLs)^23–25^, or the canalization of Boolean functions^26,27^, among others^3,28^. Despite their importance and feasibility, benchmark BN models have not yet been proposed, which makes their construction desirable.

To address this limitation, in this work, we propose benchmark BN models for GRNs^29^. These networks are built considering the structural and dynamical properties and motifs empirically observed in the BN models of GRNs^3^. Specifically, we construct four signed network models to capture specific features of four major biological kingdoms: animals, bacteria, fungi, and plants. Our work provides a solid base for testing algorithms and enabling systematic comparison of BNs.

## Methods

We construct the benchmark BNs^29^ by incorporating recurrent properties and motifs present in the GRNs as reported in prior meta-analytical studies^3,21– 28,30–35^. To do that, first, we present some necessary definitions and preliminaries. Secondly, we examine the methodology for constructing the benchmark BN models. Finally, we discuss the possible limitations of our approach.

### Definitions and preliminaries

A simple, connected, directed graph *G* = (*V*, *E*) is given by its *node set V* = {1, 2, …, *n*} and its *edge set E*, where (*u*, *v*) ∈ *E* if there is a *directed edge* or *arc* from *u* ∈ *V* to *v* ∈ *V*. For every node *u* ∈ *V*, *x*_*u*_ ∈ {0, 1} is its *state variable* or *state value*. A *configuration* is a vector of state values for all entities ***x*** = (*x*_1_, …, *x*_*n*_) ∈ {0, 1}^*n*^ at a given time. Then, a (synchronous) *Boolean network* (BN) is a discrete dynamical system defined by the map

$${\boldsymbol{F}}=({F}_{1},\ldots ,{F}_{n}):{\{0,1\}}^{n}\to {\{0,1\}}^{n},\qquad {\boldsymbol{F}}({\boldsymbol{x}})=({F}_{1}({\boldsymbol{x}}),\ldots ,{F}_{n}({\boldsymbol{x}})),$$

where *F*_*u*_ is the (Boolean) *local update function* associated with the node *u* ∈ *V*, whose expression only depends on the state values of the neighbors of *u* in the graph *G* = (*V*, *E*).

Let ***a*** = (*a*_1_, …, *a*_*n*_) ∈ {0, 1}^*n*^ be a configuration of the system. When ***F***^*m*^(***a***) = ***a*** and ***F***^*k*^(***a***) ≠ ***a*** for every positive integer *k* < *m*, we say that ***a*** is an *attractor* of length *m*. If *m* = 1, then ***a*** is called a *steady state* or a *fixed point*. Attractors of length *m* > 1 are usually referred to as *m*-*limit cycles* or *m*-*periodic orbits*. Since the system is finite, the iteration of ***F*** eventually leads to an attractor, which makes them central for studying the dynamics of BNs.

An edge is called a *self-loop* if it connects a node to itself. A *path* is a finite sequence of edges. The *in-degree *${k}_{v}^{{\rm{in}}}$ of a node *v* ∈ *V* is the number of its incoming edges (i.e., its regulatory inputs), while the *out-degree *${k}_{v}^{{\rm{out}}}$ is the number of its outgoing edges (i.e., its targets). The *average connectivity* of a network ⟨*k*⟩ is the mean in-degree of a node, i.e., the average number of regulators per node^3^. GRNs are typically *scale-free networks*, meaning that they exhibit a heavy-tailed *power-law distribution* for the out-degree^3,11,21,28,30^, that is,

$${P}_{{\rm{out}}}(k)\propto {k}^{-\gamma },\qquad \,{\rm{being}}\,k\ge {k}_{\min }^{{\rm{out}}}\quad \,{\rm{and}}\,\quad \gamma  > 1,$$

where ${k}_{\min }^{{\rm{out}}}$ is the minimum out-degree among all nodes. On the other hand, the in-degree distribution of GRNs approximates a *Poisson distribution*^3^.

Signs can be assigned to the edges in *G* = (*V*, *E*) to obtain a *signed interaction graph* (*G*, *σ*), which represents the activating and inhibiting dependencies in BNs as defined by the local update functions. That is, each edge (*u*, *v*) ∈ *E* has a sign *σ*(*u*, *v*) = + 1 if *u* activates *v*, and *σ*(*u*, *v*) = − 1 if *u* inhibits *v*. Equivalently, the sign indicates whether the local update function *F*_*u*_ is increasing (positive) or decreasing (negative) with respect to *x*_*v*_. Additionally, for a path of nodes *π* ≡ *u*_0_∣*u*_1_∣…∣*u*_*m*_, *u*_*i*_ ∈ *V*, the *path sign*, *σ*(*π*), is the product of the signs of its edges.

The signed interaction graph of a BN can be well-defined when all local update functions are *regulatory*, i.e., every variable appears once (positive or negative) in their disjunctive normal form. Aside from regulatory ones, canalizing Boolean functions are recurrent in modeling GRNs, since canalization promotes robustness to perturbations^3,26,27^. A Boolean function *F* is *canalizing* if there exists a canalizing variable *x*_*u*_, a canalizing input *a* ∈ {0, 1}, and a canalized output *b* ∈ {0, 1} satisfying

$$F({x}_{1},\ldots ,{x}_{n})=\{\begin{array}{ll}b, & \,{\rm{if}}\,{x}_{u}=a,\\ {H}_{1}({x}_{1},\ldots ,{x}_{u-1},{x}_{u+1},\ldots ,{x}_{n})\ne b, & \,{\rm{otherwise}}.\end{array}$$

If the function *H*_1_ is also canalizing, then *F* is *2-canalizing*, and, in general, assuming the previous notation, if *H*_1_, …, *H*_*k*_ are canalizing, then *F* is *k* + 1-*canalizing*, and *k* + 1 is said to be the *canalizing depth* of *F*. For a *fully canalizing function*, the canalizing depth matches the in-degree.

**Fig. 1:**
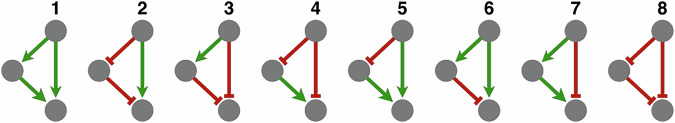
Types of coherent (1-4) and incoherent (5-8) FFLs.

A *feed-forward loop* (FFL) in a directed graph *G* = (*V*, *E*) is a connected subgraph of three nodes *u*, *v* and *w* such that (*u*, *v*), (*v*, *w*) and (*u*, *w*) belong to *E*. That is, node *u* regulates *w* directly through the edge (*u*, *v*) and indirectly through the path *u*∣*v*∣*w*. When the signs of the interactions are considered, a FFL can be classified as *coherent*, if the signs of the direct edge (*u*, *v*) and the indirect path *u*∣*v*∣*w* coincide, indicating that both have the same regulatory influence; or *incoherent*, if the signs differ, meaning that they have opposing effects. There are 2^3^ = 8 possible sign combinations for a FFL, four of them coherent and the other four incoherent. All eight FFL types are shown in Fig. 1.

**Fig. 2:**
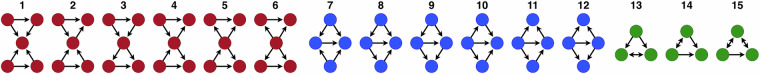
Types of FFL clusters.

FFLs that share nodes or edges form a *cluster*^3,30^. Six possible clusters arise when considering two FFLs that share one node (Motifs 1–6 in Fig. 2). Six additional clusters are obtained when considering two FFLs that share two nodes (Motifs 7–12). For clusters with three nodes, there are two possible options composed of two FFLs (Motifs 13–14) and an additional one, consisting of three FFLs (Motif 15).

A *feed-back loop* (FBL) of length *m* + 1 is a closed path *u* = *u*_0_|*u*_1_|…|*u*_*m*_ = *u* in *G* of *m* + 1 distinct nodes, i.e., a directed *cycle*. Since FBLs are paths, they can be positive or negative depending on whether the product of the signs of their edges is positive or negative. Positive FBLs are typically associated with steady-state attractors, while negative FBLs are generally linked to attractors of greater length^31^.

### Benchmark BNs construction methodology

To build the proposed benchmark BNs, we constructed a unique synthetic unsigned network of size *n* = 20 that reproduces observed metrics of essential properties and motifs of GRNs^3^. This network, which we call the *underlying network*, is used as the base topology for all the signed benchmark networks across different kingdoms. To construct it, we first generated random directed graphs and iteratively adjusted them to meet empirical metrics. Specifically, in each iteration, we examined the out-degree, in-degree, and FFL cluster distributions of the generated graphs, as well as the average connectivity. Then, we applied automated edge modifications to approximate empirical distributions.

Secondly, starting from the underlying network, we constructed four benchmark signed networks to represent the specific characteristics of the four major biological kingdoms: animals, bacteria, fungi, and plants. Signs, representing activating or inhibitory influences, were assigned to reproduce observed distributions of sign-based motifs^3^. In particular, these include the distribution of coherent and incoherent FFLs, the ratio of negative to positive interactions relative to in-degree, and the overall distribution of FBL signs.

More specifically, for each of the four kingdoms, random sign assignments are applied to the edges forming FFLs to generate 25 partially signed network candidates that best match the observed distributions of coherent and incoherent FFLs^3^. This step is performed first since not all edges belong to FFLs in the underlying network.

Each partially signed network is then used as a seed to build (totally) signed graphs by fitting the remaining sign-based motif distributions: the fraction of positive FBLs by cycle lengths 1 to 6, and the proportion of positive inputs per in-degree. To this end, a search algorithm is used that randomly assigns signs to free edges (those that do not belong to any FFL) until *M* edges remain. In parallel, *L* edges from FFLs are randomly selected according to their importance, measured by frequency of occurrence in FFLs, with less frequent edges being more likely to be chosen. Exhaustive search is then performed over the remaining *M* + *L* edges. The network with the smallest weighted error across the three target motif distributions is selected (with weights *w*_FFL_ = *w*_FBL_ = *w*_indeg_ = 1/3).

The previous routine is repeated for 1000 iterations, and different values of *M* and *L*, and the best-fitting network is selected for each seed. Finally, the optimal networks for each seed are compared, and the overall best-fitting network is chosen.

Lastly, to construct the BNs for each kingdom, we assigned a canalizing Boolean update function to every node in the benchmark signed network, excluding the external parameters. We used fully canalizing Boolean functions, as these are known to dominate in GRNs^3^. Specifically, we defined three alternative models based on the types of fully canalizing local update functions used in them. The first model uses only the logical OR and NOT operators, which corresponds to a so-called OR-NOT BN^12,32^. The second model uses, for each local function, either OR and NOT, or AND and NOT, which corresponds to a so-called AND-OR-NOT BN^13,33,34^. The third model is a network in which all local functions are canalizing and combine the operators AND, OR, and NOT^27,35^.

### Limitations

Our proposed benchmark BN models capture common structural and dynamical properties observed across many GRNs but do not reproduce the mechanistic details of any specific GRN. Therefore, they do not replace testing on real GRNs, but rather provide valid and practical complementary models for computational evaluation and comparison.

## Data Records

The dataset^29^ contains four benchmark signed network models, one for each of the biological kingdoms–animals, bacteria, fungi and plants. The underlying network is also provided.

The dataset is organized in five subfolders. One subfolder contains the adjacency matrix of the underlying topology, provided as a CSV file, together with a visualization of the network. The remaining four subfolders are named according to the respective kingdom and contain the customized network models. Each of these folders contains (i) a CSV file with the adjacency matrix of the kingdom-specific network, (ii) a visualization of the network, and (iii) three TXT files specifying different Boolean network update rules.

The complete dataset is available at 10.5281/zenodo.17406797.

## Data Overview

**Fig. 3:**
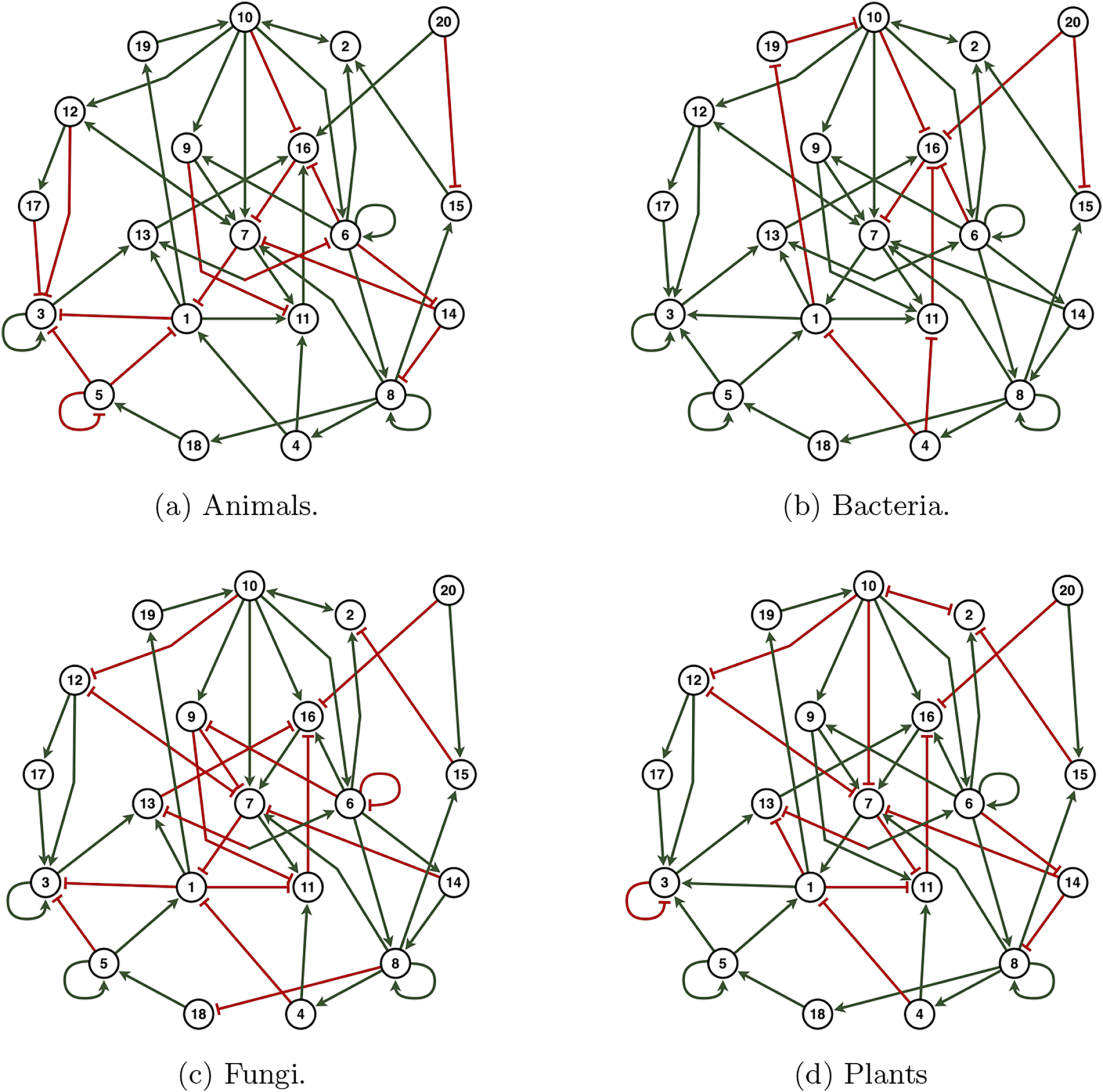
The benchmark signed networks for each of the four kingdoms. A green arc (pointed end) indicates an activating interaction, and a red arc (flat end) an inhibitory interaction. Bidirectional edges are green if both interactions are activating, red if both are inhibitory, and mixed if one is activating and the other inhibitory.

As stated before, an underlying (unsigned) network is used as the base topology for the benchmark networks of all kingdoms. The underlying network comprises 19 interacting entities and one external parameter, and is represented by a graph with 20 nodes and 50 directed edges. With the underlying topology of the underlying network, four distinct benchmark signed networks are proposed, one for each kingdom, which are shown in Fig. 3.

Lastly, for each benchmark signed network, three distinct benchmark BN models are proposed: an OR-NOT BN, an AND-OR-NOT BN, and a general canalizing BN. All models have different attractors and attractor lengths^36^ as displayed in Table 1.

**Table 1:** Type and number of attractors in the benchmark Boolean network models for every kingdom and Boolean update rule set.

Animal Kingdom					
OR-NOT		AND-OR-NOT		General canalizing	
Type	Number	Type	Number	Type	Number
Fixed point	4	*	*	Fixed point	2
*	*	2-periodic	2	2-periodic	2
*	*	*	*	4-periodic	4

Bacteria Kingdom					
OR-NOT		AND-OR-NOT		General canalizing	
Type	Number	Type	Number	Type	Number
Fixed point	2	Fixed point	6	Fixed point	10
*	*	*	*	2-periodic	4

Funghi Kingdom					
OR-NOT		AND-OR-NOT		General canalizing	
Type	Number	Type	Number	Type	Number
Fixed point	6	Fixed point	2	Fixed point	5
*	*	2-periodic	4	*	*
*	*	*	*	3-periodic	8

Plant Kingdom					
OR-NOT		AND-OR-NOT		General canalizing	
Type	Number	Type	Number	Type	Number
Fixed point	2	Fixed point	6	Fixed point	6

For OR-NOT update rules, only the Boolean functions OR and NOT are used. For AND-OR-NOT rule sets, each rule consists either exclusively of AND and NOT or exclusively of OR and NOT functions. Canalizing rules may use any combinations of AND, OR and NOT but still have to be canalizing. The type of an attractor defines if it is a fixed point or a periodic attractor with length *l*. * indicates that there are no attractors of a certain length.

## Technical Validation

The underlying network has *n* = 20 nodes, which makes it tractable for analysis using current software tools^36^. The network has one external parameter, matching that empirically observed for networks of similar size^3^. Additionally, the underlying network exhibits an average connectivity of *μ*_⟨*k*⟩_ = 2.5, consistent with the observed mean average connectivity *α*_⟨*k*⟩_ = 2.56 for comparable models^3^.

**Fig. 4:**
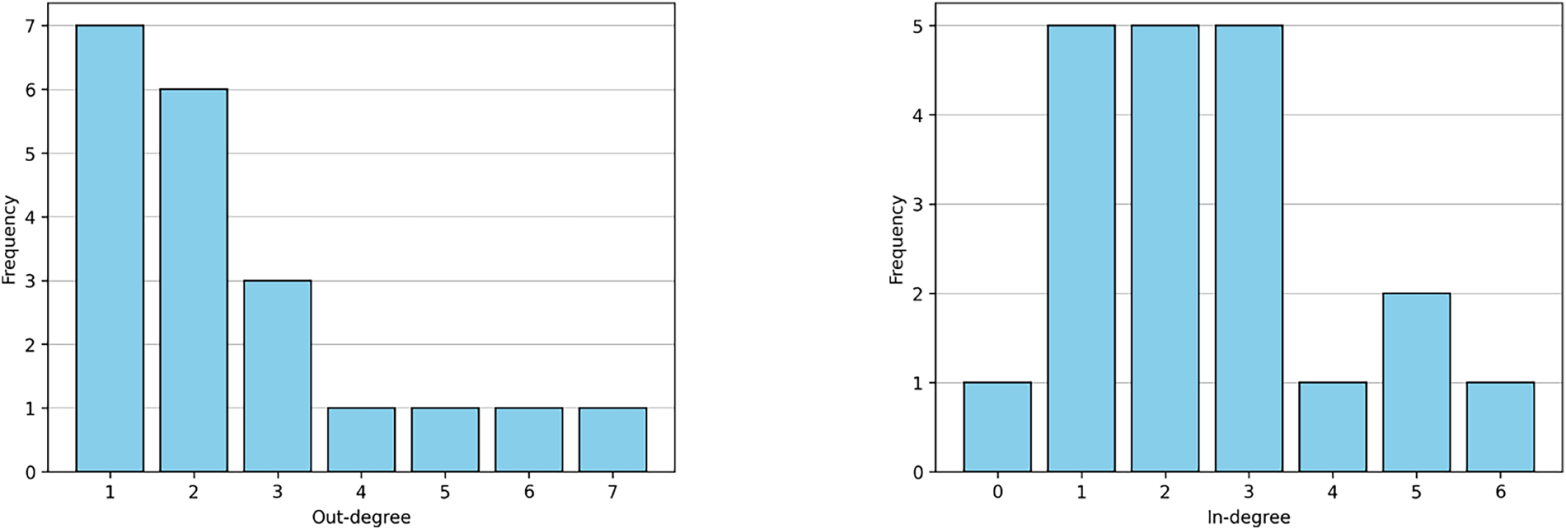
The out-degree distribution (left) and in-degree distribution (right) of the underlying network.

Network models of GRNs typically exhibit scale-free out-degree distributions, characterized by a few highly connected hub nodes and many nodes of low connectivity^3,21,22^. The underlying network follows this pattern and is consistent with power-law distribution (although small in number of nodes for significant statistical testing). The out-degree distribution is shown in Fig. 4 (left), along with the in-degree distribution (right).

**Fig. 5:**
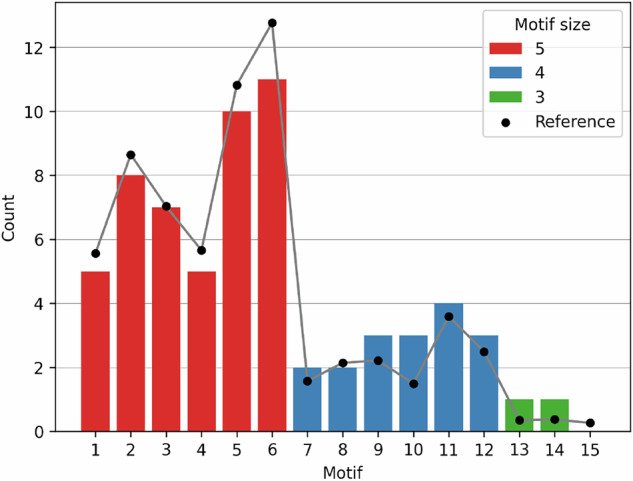
Frequency distribution of FFLs in the underlying network and distribution of relative frequencies in real GRN models^3^.

As mentioned previously, one of the main subgraph motifs in GRNs are FFLs^23–25^. FFLs usually form clusters, where the same edge belongs to multiple FFLs (see Figure 2). Such clusters are independent of the interaction signs and can therefore be analyzed for the underlying network. The distribution of FFL clusters in the underlying network approximates that observed for real GRN models^3^, as shown in Fig. 5.

### Benchmark signed network model per kingdom

**Fig. 6:**
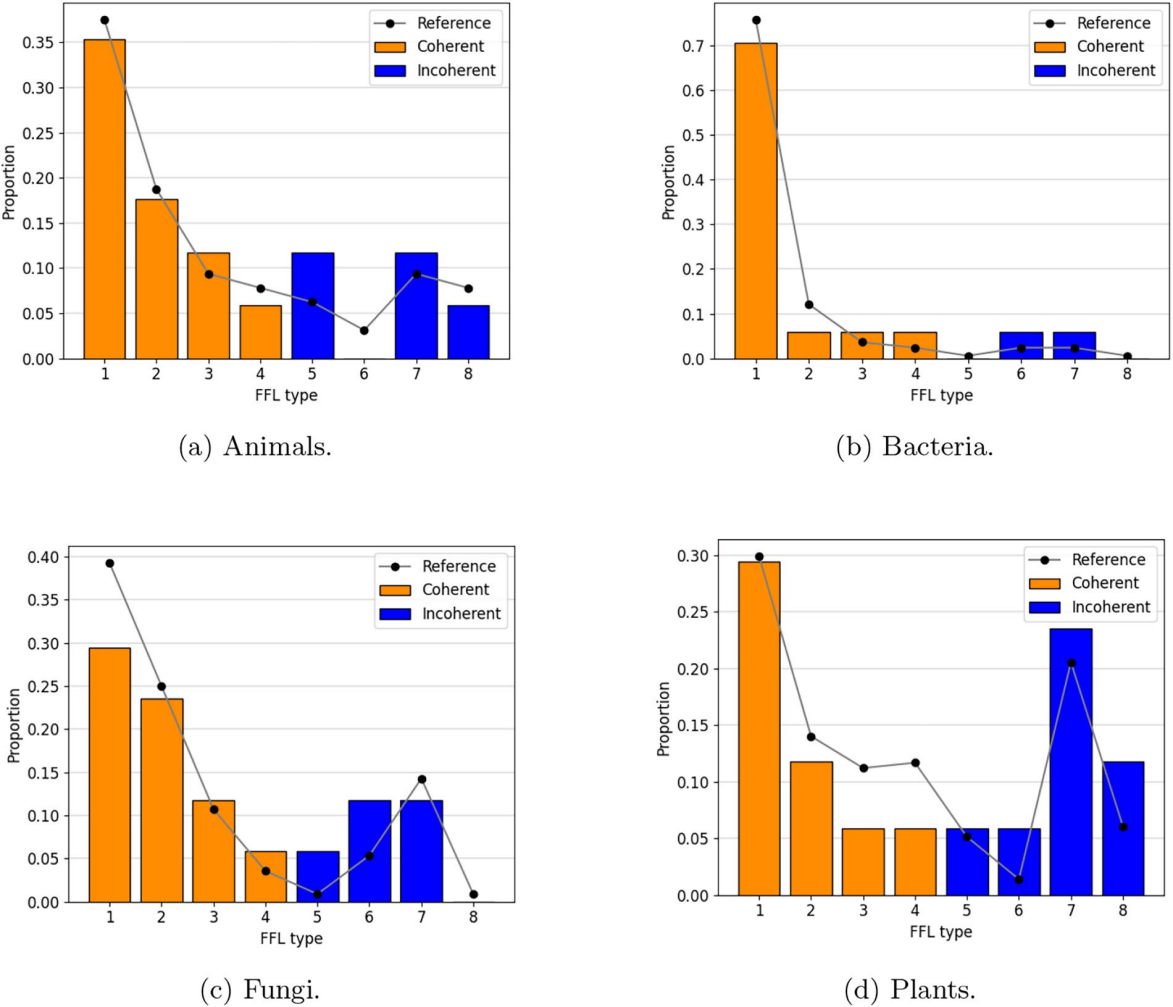
Distribution of FFL types in the benchmark signed networks and corresponding distribution in real GRN models^3^ across kingdoms.

The assignment of a positive or negative sign to each of the three edges of a FFL (corresponding to an activating or an inhibitory interaction) yields 2^3^ possible types. By considering this, FFLs can be classified as coherent or incoherent^3,23,37^ (see Figure 1). The distribution of FFL types in the benchmark signed networks matches that empirically observed in real GRN models^3^, which are shown in Fig. 6.

**Fig. 7:**
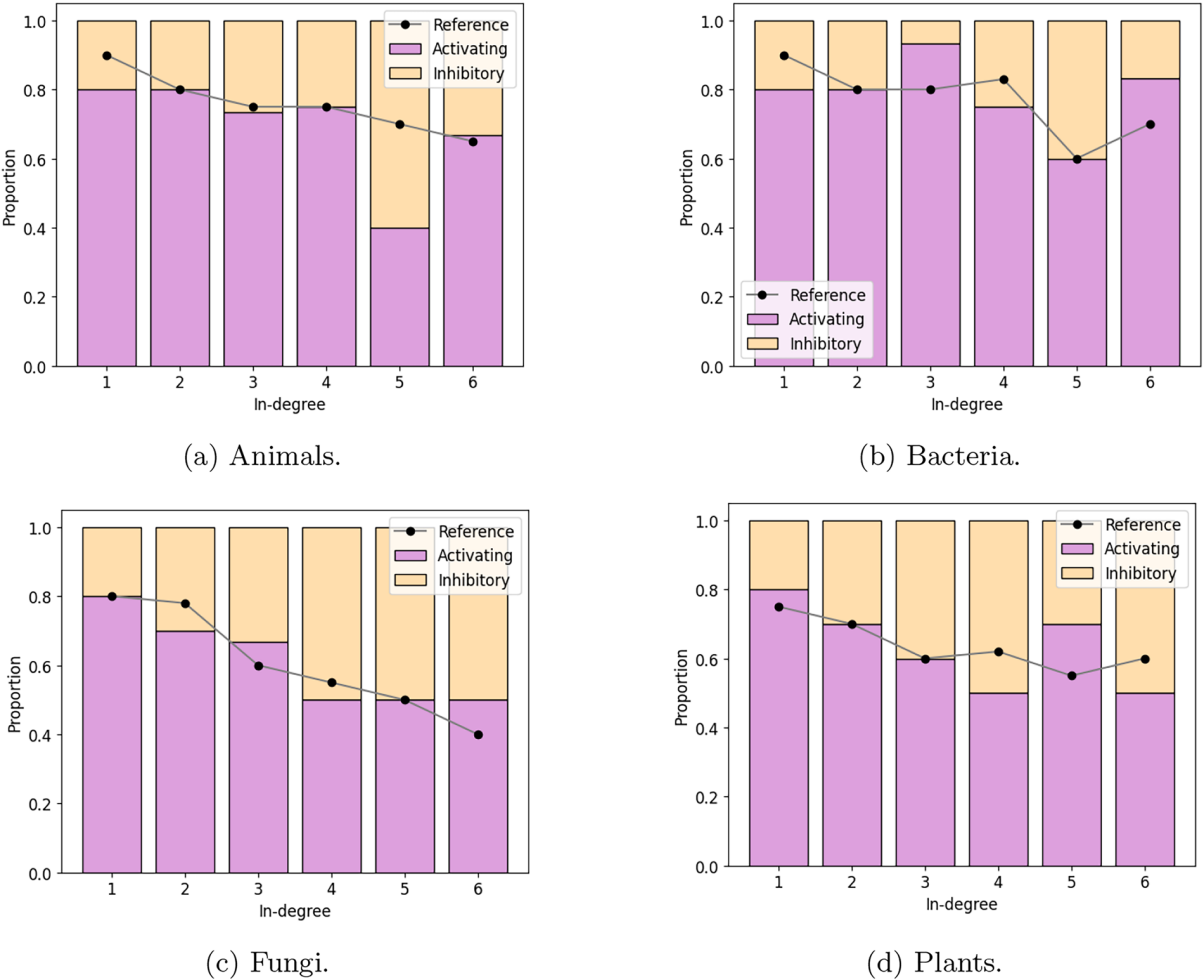
Distribution of activating vs inhibitory interactions by in-degree in the benchmark signed networks and corresponding distribution in real GRN models^3^.

The proportion of activating to inhibitory inputs by in-degree for each kingdom has also been analyzed in real GRN models^3^. Each of the proposed benchmark BN models reproduces this corresponding proportion, as shown in Fig. 7.

**Fig. 8:**
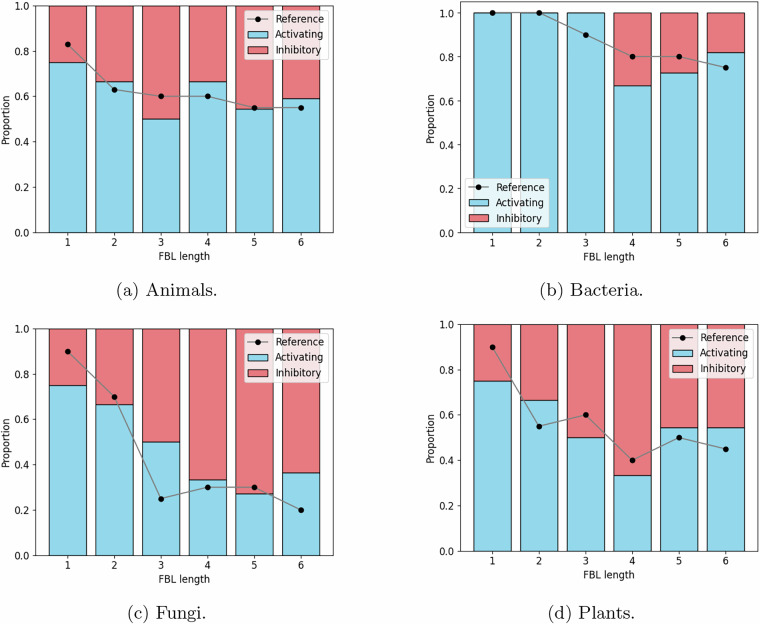
Distribution of activating vs inhibitory interactions by FBL length in the benchmark signed networks and corresponding distribution in real GRN models^3^ across kingdoms.

Lastly, the proportion of activating vs inhibitory edges has also been analyzed for FBLs of different sizes^3^, showing that FBLs are enriched for inhibitory edges. Each benchmark BN model reproduces this pattern, as shown in Fig. 8.

### Benchmark Boolean network models per kingdom

Different local update functions can produce very different dynamics for the same signed network. To account for this, for each benchmark signed network, we propose three benchmark BN models per kingdom, all sharing the same signed graph, but with different canalizing local update functions. All local update functions are chosen so that the canalization depth equals the number of inputs of each node, in accordance with empirical observations in real GRN models^3,26,27^. As previously stated, the first model corresponds to an OR-NOT BN^12,32^, the second model to an AND-OR-NOT BN^13,33,34^, and the third corresponds to a general canalizing BN^27,35^. The dynamics of the models range from fixed points to coexisting attractors of greater length, showing how tuning the local update functions can generate diverse behaviors and capture different real-world scenarios. The proposed models and associated dynamical analyzes are provided in 10.5281/zenodo.17406797.

## Data Availability

The dataset containing our Benchmark GRNs is available at 10.5281/zenodo.17406797.

## Code availability

The code to assign signs to the underlying network while matching predefined distributions is provided at 10.5281/zenodo.17406797.
